# Impact of prior endoscopic or surgical interventions on clinical outcomes after peroral endoscopic myotomy for achalasia

**DOI:** 10.1007/s00464-025-12258-3

**Published:** 2025-10-06

**Authors:** John DeWitt, Sarah Stainko, Anthony Perkins, Michael Rosenheck, Mohammad Al-Haddad

**Affiliations:** 1 Department of Gastroenterology and Hepatology, Indiana University Health Medical Center, 550 N. University Blvd., UH 4100, Indianapolis, IN 46202 USA; 2 Department of Biostatistics, Indiana University Health Medical Center, Indianapolis, IN USA

**Keywords:** Peroral endoscopic myotomy, Achalasia, Heller myotomy, Pneumatic dilation, Botox injection, Clinical outcomes

## Abstract

**Background:**

The aim of this study is to compare outcomes of POEM for achalasia between those with (PI) or without (NPI) previous disease intervention.

**Methods:**

Single-center retrospective study of consecutive achalasia patients with or without ≥ 1 prior intervention with pneumatic dilation (PD), Heller myotomy (LHM), and/or Botox injection (BTI) who underwent POEM and had ≥ 6-month follow-up. Baseline testing: Eckardt Score (ES), high-resolution manometry (HRM), and functional lumen imaging probe (FLIP) of the esophagogastric junction (EGJ) at 50-mL distention. Between 6 and 12 months after POEM, patients were questioned about daily PPI use and HRM, ES, FLIP, EGD, and pH testing off anti-secretory medications were repeated when possible. Clinical response was defined as follows: ES ≥ 3, EGJ-DI > 2.8 mm^2^/mmHg, and integrated relaxation pressure (IRP) < 15 mmHg. GERD was defined as acid exposure time (AET) > 6%. Outcomes were compared between the PI and NPI groups.

**Results:**

471 patients (mean: 55 ± 19 yrs; 60% M) with type 1 (17%), 2 (72%) or 3 (11%) achalasia with no prior (*n* = 325) or a prior (*n* = 126) intervention were identified. The PI group was older (*p* < 0.001), had a lower baseline ES (*p* = 0.03), lower IRP (*p* = 0.001), higher EGJ-DI (*p* = 0.001), and a longer POEM procedure time (*p* = 0.001) compared to the NPI group. Mean overall clinical response by ES (95.3%), IRP (86.7%), and EGJ-DI (86.2%), and daily PPI use (30%), esophagitis (65%), and AET > 6% (49%) were similar between the two groups.

**Conclusion:**

Achalasia patients with previous LES-directed interventions undergoing POEM are older, have lower ES, lower LES pressure/tone, and require longer POEM times compared to those without previous interventions. Prior intervention does not impact the frequency of clinical response, daily PPI use, esophagitis or post-POEM GERD (ClinicalTrials.gov NCT02770859).

Achalasia is an esophageal motility disorder characterized by aperistalsis, incomplete relaxation of the lower esophageal sphincter (LES), and elevated integrated relaxation pressure (IRP) on high-resolution esophageal manometry (HRM) [1]. Treatment traditionally focuses on relaxation, disruption or incision of the lower esophageal sphincter (LES) by botulinum toxin injection (BTI), pneumatic dilation (PD) or laparoscopic Heller myotomy (LHM). However, these techniques may have limited efficacy or lose response over time thus necessitating retreatment with the same or a different method [2]. Peroral endoscopic myotomy (POEM) is an endoscopic technique for incision of the LES and/or suprasphincteric esophagus and has reported efficacy for treatment of achalasia even after prior failed intervention (PI). A meta-analysis of 8 studies between 2013 and 2020 enrolling 1797 patients found that technical success, clinical success, and adverse events of POEM for achalasia were similar between those with or without previous intervention [3]. However, previous studies evaluating the impact of previous intervention on achalasia outcomes after POEM are often limited by small cohorts, comparison of treatment by only one technique, or evaluation of few clinically relevant variables. In this large retrospective single-center study, we compared the safety and multiple clinically relevant outcomes of POEM for achalasia in patients with or without previous disease intervention. We hypothesized that prior intervention would not impact on the efficacy or safety of POEM in this population and that the frequency of prior intervention in our population would decrease over time as use of POEM became more widely adopted locally.

## Materials and methods

### Patient selection and study design

This IRB-approved study retrospectively reviewed prospectively collected data (ClinicalTrials.gov number, NCT02770859) from consecutive patients who underwent POEM for type 1, 2, or 3 achalasia at Indiana University Health Hospital in Indianapolis between 2014 and 2022. Patients were categorized into those who did (PI group) or did not (NPI group) receive ≥ 1 prior intervention with pneumatic dilation (PD), laparoscopic Heller myotomy (LHM), and/or Botox injection (BTI). Patients were excluded if there was a non-achalasia diagnosis, absent/withdrawn research consent, previous POEM, or did not have at least 6 months of follow-up after POEM. Written informed consent was obtained from all patients to permit prospective data collection used for the study. All authors had access to the study data and reviewed and approved the final manuscript. Study methods and results when possible were reported according to the recommendations from the Standards for Reporting of Diagnostic Accuracy Studies (STARD).

### Pre-procedure evaluation

Demographic information and patient-reported frequency of dysphagia, chest pain, regurgitation, and weight change were recorded to calculate an Eckardt score (range: 0–12, with higher score indicating more severe disease) [4]. Baseline EGD, HRM, and FLIP of the esophagogastric junction (EGJ) was performed.

### High-resolution esophageal manometry (HRM)

Manometry was performed in the left lateral position with 30-degree head-elevation. A 4.2-mm, solid-state, manometry-impedance catheter with 36 circumferential pressure sensors at 1-cm intervals and 18 impedance channels (Medtronic, Minneapolis, MN) was placed transnasally and positioned with the pressure sensors located from the hypopharynx, esophagus and 3–5 cm into the stomach. Once the catheter was in position and following an acclimation period, baseline resting EGJ morphology and pressure was assessed during normal respiration without swallows. Ten 5-mL liquid swallows were then performed in the supine position. Additional upright swallows were performed as required. Study swallows were analyzed using the ManoView version 3.0 analysis software (Medtronic Minneapolis, MN) and Chicago Classification version 3.0 or 4.0 depending on date of testing [5, 6].

### EGD and FLIP procedures

EGD and FLIP were performed in the left lateral position under propofol sedation. With the endoscope withdrawn, an 8-cm EF-325N or 16-cm EF-322N FLIP (EndoFLIP®, Medtronic, Inc., Shoreview, MN) catheter was initially calibrated to atmospheric pressure and passed transorally across the EGJ to ensure that at least two sensors were in the stomach. Repeat EGD was done to confirm the balloon had traversed the EGJ and was positioned at the midpoint of the impedance sensors prior to inflation. Controlled volumetric distension of the balloon to 50 mL was performed for at least 30 s, while measurements of EGJ cross-sectional area, intrabag pressure, and EGJ diameter were assessed. If bag migration was suspected or peristalsis occurred, the bag was repositioned and the measurement repeated. The EGJ distensibility index (EGJ-DI) at  50mL distension volume was calculated [7].

### POEM procedure

All POEM procedures were performed by two gastroenterologists with patients under general anesthesia. For type 1 and 2 achalasia patients, approximately 8–10 cm proximal to the EGJ, a mixture of saline with methylene blue was injected into the submucosa. After adequate lift, a longitudinal 2–3 cm mucosal incision (ENDO CUT® I, Effect 2, Cut Duration 2, Cut Interval 1) was made with a Hybrid T knife (ERBE USA, Inc., Marietta GA) using an electrosurgical generator (ERBE VIO® 300D, ERBE USA, Inc., Marietta GA). A submucosal tunnel was created and through careful dissection extended (Forced Coag™, Effect 2, maximum 50 watts) about 2–3 cm into the gastric cardia. Bleeding or potentially bleeding sites were treated with Coagulation hemostatic forceps (Olympus America, Center Valley, PA) using soft coagulation (Effect 5, 80 Watts) current. A myotomy was performed using ENDO CUT® I as above or Spray Coagulation (Effect 2, 35 Watts) in a proximal-to-distal direction and extended approximately 2 cm into the gastric cardia. After myotomy, the entry of the tunnel was then closed with endoscopic clips (Resolution™, Boston Scientific, Inc., Marlborough, MA) and/or endoscopic suturing (Overstitch, Apollo Endosurgery, Austin TX). For type 3 achalasia patients, the procedure performed described above, except the length of myotomy was tailored to length of spastic segments noted on HRM. Orientation of the submucosal tunnel (i.e., anterior vs. posterior), type of myotomy (circular alone vs. full thickness), and total myotomy length were recorded.

### Follow-up after POEM

Between 6 and 12 months after POEM, all patients were offered same day HRM, symptom assessment for ES score, EGD, FLIP of the EGJ, and ambulatory wireless pH testing [8]. When all tests were feasible, HRM was performed initially in an unsedated patient followed 1–2 h later by EGD under propofol sedation for FLIP measurement and placement of the wireless capsule. Prior to placement, the Bravo™ pH capsule (Medtronic, Inc., Shoreview, MN) was calibrated, the delivery system was passed transorally, positioned 6 cm above the EGJ and deployed.

When all tests were not performed in the same day, all were completed within a 1-month timespan. Evaluations and diagnostics were performed after cessation of anti-secretory therapy for at least 1 week. The severity of any endoscopic esophagitis was classified by the Los Angeles Classification [9]. Adverse events related to POEM were graded by the classification described by Werner et al. [10]. The following were not considered adverse events: (1) easily controlled bleeding, (2) incidental radiologic findings (i.e., pneumoperitoneum), (3) superficial esophageal mucosal injuries that did not require closure, and (4) post-POEM hypoxemia or change in vital signs without end-organ effects (i.e., myocardial infarction).

### Definitions

Technical success was completion of the intended length of myotomy during POEM. Clinical response after POEM was defined by three independent metrics in the absence of a severe procedure-related adverse event or requirement for repeat LES-directed intervention: Eckardt Score ≤ 3 [4], EGJ-DI > 2.8 mm^2^/mmHg [11], or IRP < 15 mmHg [5]. GERD was defined as total test duration acid exposure time > 6% [12].

### Statistical analysis

Baseline findings, results of the POEM, post-procedure clinical response, PPI use, esophagitis grades, and results of clinical testing were compared between those with or without previous intervention before POEM. The frequency of patients with or without previous intervention expressed as a percentage of total POEMs performed annually during the study period was also calculated. Continuous variables are described as means ± standard deviations or medians with ranges. Dichotomous variables are described as proportions. Two sample *T* tests for continuous outcomes and Fisher’s exact test for categorical variables were performed to test differences between the two groups. A *p* value < 0.05 was used to determine significance. All analyses were performed using SAS v9.4 (Cary NC, USA).

## Results

### Study population

During the study period, 721 patients underwent POEM and 289 (40%) with a non-achalasia diagnosis (*n* = 233), absent/withdrawn research consent (*n* = 50), or previous POEM (*n* = 4) were excluded. The study population included 471 patients (mean age 55 ± 19 yrs.; 60% male) with type 1 (17%), type 2 (72%) or type 3 (11%) achalasia (Table 1). Mean baseline Eckardt score and IRP were 7.7 ± 2.0 and 30 ± 13 mmHg, respectively. Previous intervention (PI) was performed in 126 (27%) and included BTI alone (*n* = 80), LHM alone (*n* = 23), PD alone (*n* = 13), or multiple procedures (*n* = 10). The remaining 325 (73%) had no prior intervention (NPI) for achalasia. During POEM, a mean 8.9 ± 3.3 cm myotomy was performed. Overall adverse and serious adverse events were 3.0% and 1.9%, respectively.

**Table 1:** Baseline and procedure characteristics for 471 consecutive patients with achalasia with (*n* = 126) or without (*n* = 345) previous endoscopic or surgical intervention(s) prior to POEM

	Overall (*n* = 471)	Prior intervention (*n* = 126)	No prior intervention (*n* = 345)	*P* value
Baseline characteristics				
Mean (SD) Age, yrs	54.6 (18.5)	60.0 (18.1)	52.7 (18.3)	< 0.001
Mean (SD) BMI, kg/m^2^	28.3 (7.1)	27.8 (6.3)	28.5 (7.3)	0.366
Male, *n* (%)	281 (59.7)	71 (56.4)	210 (60.9)	0.397
Achalasia type, *n* (%)				0.087
1	80 (17.0)	26 (20.6)	54 (15.6)	
2	341 (72.4)	82 (65.1)	259 (75.1)	
3	50 (10.6)	18 (14.3)	32 (9.3)	
Mean (SD) Eckardt score	7.7 (2.0)	7.2 (2.3)	7.9 (1.9)	0.026
Mean (SD) IRP (mmHg)	29.8 (12.7)	27.1 (14.1)	30.8 (12.1)	0.001
Mean (SD) EGJ-DI 50 mL(mm^2^/mmHg)	1.05 (1.00)	1.45 (1.48)	0.92 (0.73)	0.002
Prior intervention (*n*, %)		126 (26.8)		
Botox injection (BTI)		80 (63.5)		
Laparoscopic Heller myotomy (LHM)		23 (18.2)		
Pneumatic dilation (PD)		13 (10.3)		
LHM and BTI		5 (4.0)		
LHM and PD		4 (3.2)		
PD and BTI		1 (0.8)		
POEM procedure				
Technical success (%)	99.2	99.2	99.4	1.0
Mean (SD) myotomy, cm	8.9 (3.3)	9.3 (3.5)	8.8 (3.2)	0.210
Mean procedure time (SD)	73.6 (32.5)	81.7 (35.7)	70.6 (30.9)	0.001
Myotomy orientation, *n* (%)				0.220
Right anterior	17 (3.6)	6 (4.8)	11 (3.2)	
Anterior	38 (8.1)	15 (11.9)	23 (6.7)	
Posterior	401 (85.3)	103 (81.8)	298 (86.6)	
Right lateral	5 (1.1)	0 (0.0)	5 (1.4)	
Left lateral	9 (1.9)	2 (1.6)	7 (2.0)	
Overall adverse event, *n* (%)	14 (3.0)	4 (3.2)	10 (2.9)	0.88
Serious adverse event, *n* (%)	9 (1.9)	2 (1.6)	7 (2.0)	0.76

*BMI* body mass index, *BTI* botulinum toxin injection, *LHM* laparoscopic Heller myotomy, *PD* pneumatic dilation, *POEM* peroral endoscopic myotomy, *EGJ*-*DI* esophagogastric junction distensibility index, *IRP* integrated relaxation pressure

The PI group was older (mean 60 ± 18 vs. 53 ± 18 yrs, *p* < 0.001), had a lower baseline ES (mean 7.2 ± 2.3 vs. 7.9 ± 1.9; *p* = 0.03), lower IRP (mean 27 ± 14 vs. 31 ± 12 mmHg, *p* = 0.001), and higher EGJ-DI (1.5 ± 1.5 vs. 0.9 ± 0.7 mm^2^/mmHg, *p* = 0.002) compared to the NPI group (Table 1). Mean procedure time was longer in the PI compared to the NPI group (82 ± 36 vs. 71 ± 31 min., *p* = 0.001). Otherwise, demographics, overall and serious adverse events, myotomy orientation and length were similar between the two groups.

Table 2 demonstrates the percentage of total POEMs annually during the study period who did or did not receive previous intervention for achalasia. No statistically significant differences between the two groups were noted during the 11-year period (*p* = 0.27).

**Table 2:** Frequency of patients with or without previous intervention expressed as a percentage of total POEMs performed annually during the study period

	2014 (*n* = 1)	2015 (*n* = 4)	2016 (*n* = 15)	2017 (*n* = 30)	2018 (*n* = 59)	2019 (*n* = 64)	2020 (*n* = 58)	2021 (*n* = 75)	2022 (*n* = 78)	2023 (*n* = 70)	2024 (*n* = 17)
No prior intervention	0 (0.0)	3 (75.0)	11 (73.3)	17 (56.7)	42 (71.2)	48 (75.0)	42 (72.4)	53 (70.7)	61 (78.2)	52 (74.3)	16 (94.1)
Prior intervention	1 (100.0)	1 (25.0)	4 (26.7)	13 (43.4)	17 (28.8)	16 (25.0)	16 (27.6)	22 (29.3)	17 (21.8)	18 (25.7)	1 (5.9)

*P* value = 0.269

Table 3 illustrates the technical success, clinical success and results of follow-up testing for the PI and NPI groups. Clinical response by Eckardt score was similar (94.1% vs. 95.7%, *p* = 0.56). Post-procedure HRM and FLIP were performed in 165 (35%) and 289 (61%) of the study population. There was no difference in clinical response by IRP (85.0 vs. 87.2%, *p* = 0.79) or EGJ-DI (85.5% vs. 87.4%, *p* = 0.84) between the two groups.

**Table 3:** Clinical response and follow-up testing after POEM for 471 consecutive patients with achalasia with (*n* = 126) or without (n = 345) previous endoscopic or surgical intervention(s)

Variable	Overall (*n* = 471)	Prior intervention (*n* = 126)	No prior intervention (*n* = 345)	*P* value
Clinical response (%)				
Eckardt score 0–3	95.3	94.1	95.7	0.557
IRP < 15 mmHg	86.7 (*n* = 165)	85.0 (*n* = 40)	87.2 (*n* = 125)	0.790
EGJ-DI > 2.8 mm^2^/mmHg	86.2 (*n* = 289)	85.5 (*n* = 69)	86.4 (*n* = 229)	0.843
Clinical testing, mean (SD)				
Mean (SD) Eckardt score	1.0 (1.4)	1.2 (1.8)	0.9 (1.2)	0.138
Mean (SD) IRP (mmHg)	9.2 (5.8)	9.3 (6.0)	9.1 (5.7)	0.308
Mean (SD) EGJ-DI (mm^2^/mmHg)	4.7 (2.2)	4.7 (2.1)	4.7 (2.2)	0.731
Mean (SD) DeMeester score	32.4 (30.5)	35.3 (30.8)	31.4 (30.5)	0.292
Acid exposure time > 6%	48.9 (*n* = 135)	60.6 (*n* = 33)	45.1 (*n* = 102)	0.161
Esophagitis grade	*n* = 300	*n* = 70	*n* = 230	0.677
None (*n*, %)	100 (34.7)	24 (34.3)	80/230 (34.8)	
A/B (*n*, %)	148 (49.3)	37 (52.9)	111/230 (48.3)	
C/D (*n*, %)	48 (16.0)	9 (12.9)	39/230 (17.0)	
Daily PPI use, (%)	101 (29.8) (*n* = 339)	29 (34.5) (*n* = 84)	72 (28.2) (*n* = 255)	0.275

*POEM* peroral endoscopic myotomy, *EGJ-DI* esophagogastric junction distensibility index, *IRP* integrated relaxation pressure, *PPI* proton pump inhibitor

At follow-up, EGD and pH testing were performed in 300 (64%) and 135 (29%), respectively. Esophagitis endoscopically was noted in 65.3% and was classified as LA Grade A or B in 49.3% and LA Grade C or D in 16%. By pH testing, GERD (AET > 6%) was noted in 49% and PPI use was reported by 30%. Between the PI and NPI groups, PPI use (34.5% vs. 28.2%, *p* = 0.28), severity of esophagitis (*p* = 0.68), and frequency of GERD by pH testing (60.6% vs. 45.1%, *p* = 0.16), respectively, were similar between the PI and NPI groups.

Subgroup analysis was performed to evaluate the impact of previous LHM with fundoplication on the frequency of GERD and elevated AET for patients with or without previous LES intervention who underwent post-POEM testing (Table 4). For the 32 patients with previous LHM (23 LHM alone and 9 with LHM and at least one other LES intervention), AET (*p* = 0.26) and esophagitis grade (*p* = 0.89) were similar to the other PI and NPI groups. Results were also similar (Table 5) when comparing AET (*p* = 0.18) and esophagitis grade (*p* = 0.86) between those with LHM alone (*n* = 23, other PIs (*n* = 103), and NPI groups (*n* = 345).

**Table 4:** Comparison of elevated acid exposure time and esophagitis grade for patients undergoing Laparoscopic Heller Myotomy (LHM) with or without another LES intervention (*n* = 32), patients with at least one non-LHM intervention (*n* = 94), and patients without any previous achalasia intervention (*n* = 345)

Variable	LHM (*n* = 32)	Other intervention (*n* = 94)	No prior intervention (*n* = 345)	*P* value
	*n* = 6	*n* = 27	*n* = 102	
Acid exposure time > 6%	3 (50.0)	17 (63.0)	46 (45.1)	0.255
Esophagitis grade	*n* = 19	*n* = 51	*n* = 230	0.898
None (*n*, %)	6 (31.6)	18 (35.3)	80 (34.8)	
A/B (*n*, %)	11 (57.9)	26 (51.0)	111 (48.3)	
C/D (*n*, %)	2 (10.5)	7 (13.7)	39 (17.0)

**Table 5:** Comparison of elevated acid exposure time and esophagitis grade for patients undergoing Laparoscopic Heller Myotomy (LHM) alone (*n* = 23), patients with at least one other intervention (*n* = 103), and patients without any previous achalasia intervention (*n* = 345)

Variable	Heller (*n* = 23)	Other intervention (*n* = 103)	No prior intervention (*n* = 345)	*P* value
	*n* = 5	*n* = 28	*n* = 102	
Acid exposure time > 6%	2 (40.0)	18 (64.3)	46 (45.1)	0.183
Esophagitis grade	*n* = 15	*n* = 55	*n* = 230	0.859
None (*n*, %)	4 (26.7)	20 (36.4)	80 (34.8)	
A/B (*n*, %)	9 (60.0)	28 (50.9)	111 (48.3)	
C/D (*n*, %)	2 (13.3)	7 (12.7)	39 (17.0)	

## Discussion

In this large single-center series of consecutive patients with types 1, 2, and 3 achalasia, those who received previous intervention (PI) with LHM, PD, and/or BTI prior to POEM required longer POEM times compared to those with no previous intervention (NPI). Other studies have also documented that previous endoscopic intervention is associated with longer POEM procedure times compared to treatment-naïve procedures [13–16]. It is likely that longer operative time in the PI group is attributable to increased submucosal fibrosis or a larger diameter and sigmoid configuration of the esophageal lumen in patients with long-standing disease. Endoscopists should therefore consider planning for longer POEM procedures in patients with previous endoscopic interventions.

In the current study, the PI group was older, had a lower Eckardt score, lower baseline IRP and higher EGJ-DI compared to the NPI group. We surmise that patients with recrudescence after previous intervention (i.e., the PI group) may present earlier clinically for retreatment than those with a new diagnosis and no previous intervention. Thus, objective findings at representation in the PI group in our study likely reflect continued objective benefit from previous LES intervention for achalasia. To our knowledge, this is the first study showing an objective (EGJ-DI and IRP) and subjective (Eckardt score) difference between these two groups requiring POEM for achalasia.

We found that POEM is a safe procedure without any significant difference in the incidence of overall adverse events (3%) and serious adverse events (1.9%) between the treatment-naive and previous treatment groups. This finding has also been documented by other studies [15–18]. Therefore, increasing procedure time required in patients with prior LES-directed intervention does not increase the incidence of adverse events.

We hypothesized that the adoption of POEM at our hospital for treatment of achalasia would be associated with a decreased use of other LES-directed interventions for patients with a new diagnosis of achalasia. Nevertheless, there was no change in the frequency of patients with or without previous intervention expressed as a percentage of total POEMs performed annually during the study period. With rare exception, typically between 20 and 30% of the patients undergoing POEM during our study period received previous intervention, which are values similar to [16, 17, 19] or slightly lower [15, 18] than other previous studies.

In our study, clinical response after POEM by Eckardt score was similar between the PI and NPI groups. A recent meta-analysis evaluating 8 studies in 1797 patients also concluded that clinical success after POEM was similar between treatment naive patients and those with prior intervention [3]. Among the 165 (35%) and 289 (61%) who obtained post-POEM HRM and FLIP, respectively, the measured IRP and EGJ compliance was also similar between two groups. To our knowledge, this is the first study that evaluated the impact of prior achalasia intervention on EGJ-DI and the first testing all three metrics simultaneously after POEM.

We found post-POEM GERD (acid exposure time > 6%) in 48.9% of the 135 patients who underwent pH testing and any degree of esophagitis in 65.3% of the 300 patients with follow-up endoscopy. The incidence of GERD and esophagitis were similar between the two groups. Other authors have also reported that prior intervention does not impact the incidence of esophagitis or abnormal pH testing after POEM [15, 17, 18]. Furthermore, subgroup analysis demonstrated that esophageal acid exposure time and grade of esophagitis after POEM was similar in patients with previous LHM alone (or in combination with another intervention) and patients with either another achalasia intervention or no prior intervention before POEM.

The current study represents one of the largest single-center studies [15, 16] to date which evaluates the impact of prior LES intervention on patients with achalasia undergoing peroral endoscopic myotomy. Furthermore, we assessed with impact of previous LHM, PD, and/or BTI on clinically relevant metrics, such as the Eckardt score, high-resolution manometry, FLIP, acid exposure time, esophagitis, and PPI use. Nevertheless, our study does have two important limitations. First, like other large comparative studies, follow-up EGD, high-resolution manometry, and pH testing were not obtained in the entire study cohort. Therefore, results obtained only reflect outcomes from patients in whom testing was performed. Second, although data were collected prospectively, analysis was retrospective.

In conclusion, patients with previous LES intervention for achalasia are older, have lower baseline LES pressure/tone and Eckardt scores and require longer POEM times compared to those without previous treatment. Prior intervention does not impact on the clinical response or frequency of daily PPI use, esophagitis, or post-POEM GERD in these patients.

## Abbreviations

BTI: Botulinum toxin injection
BMI: Body mass index
EGJ: Esophagogastric junction
EGJOO: Esophagogastric outlet obstruction
EGJ-DI: Esophagogastric junction distensibility index
ES: Eckardt Score
FLIP: Functional lumen imaging probe
GERD: Gastroesophageal reflux disease
HRM: High-resolution esophageal manometry
IRP: Integrated relaxation pressure
LES: Lower esophageal sphincter
LHM: Laparoscopic Heller myotomy
POEM: Peroral endoscopic myotomy
PD: Pneumatic dilation
